# ‘RetinoGenetics’: a comprehensive mutation database for genes related to inherited retinal degeneration

**DOI:** 10.1093/database/bau047

**Published:** 2014-06-17

**Authors:** Xia Ran, Wei-Jun Cai, Xiu-Feng Huang, Qi Liu, Fan Lu, Jia Qu, Jinyu Wu, Zi-Bing Jin

**Affiliations:** ^1^Institute of Genomic Medicine, Wenzhou Medical University, Wenzhou 325027, China, ^2^Division of Ophthalmic Genetics, Laboratory for Stem Cell and Retinal Regeneration, The Eye Hospital of Wenzhou Medical University, Wenzhou 325027, China and ^3^The State Key Laboratory Cultivation Base and Key Laboratory of Vision Science, Ministry of Health People’s Republic of China, Wenzhou 325027, China

## Abstract

Inherited retinal degeneration (IRD), a leading cause of human blindness worldwide, is exceptionally heterogeneous with clinical heterogeneity and genetic variety. During the past decades, tremendous efforts have been made to explore the complex heterogeneity, and massive mutations have been identified in different genes underlying IRD with the significant advancement of sequencing technology. In this study, we developed a comprehensive database, ‘RetinoGenetics’, which contains informative knowledge about all known IRD-related genes and mutations for IRD. ‘RetinoGenetics’ currently contains 4270 mutations in 186 genes, with detailed information associated with 164 phenotypes from 934 publications and various types of functional annotations. Then extensive annotations were performed to each gene using various resources, including Gene Ontology, KEGG pathways, protein–protein interaction, mutational annotations and gene–disease network. Furthermore, by using the search functions, convenient browsing ways and intuitive graphical displays, ‘RetinoGenetics’ could serve as a valuable resource for unveiling the genetic basis of IRD. Taken together, ‘RetinoGenetics’ is an integrative, informative and updatable resource for IRD-related genetic predispositions.

**Database URL:** http://www.retinogenetics.org/.

## Introduction

The retina, an essential part of brain associated with vision production and transmission, is susceptible to a variety of diseases (1). Among these diseases, inherited retinal degeneration (IRD) affects ∼1 in 2000–3000 individuals worldwide and is the leading cause of visual loss in young people (2). It includes retinitis pigmentosa, Usher syndrome, Leber congenital amaurosis, macular degeneration and other retinal disorders (3). Till now, tremendous efforts have been made on the study of the causality of IRD, and a large amount of mutations as well as IRD-related genes have been identified. It was reported that one of the most striking features of IRD is its exceptional heterogeneity, including genetic heterogeneity and clinical variety (4). Notably, a specific phenotype of IRD can be caused by mutations in different genes, and the defects in the same gene may substantially lead to different disease phenotypes (3). Because descriptions of disease phenotypes in publications were diverse from each other, understanding genotype– phenotype correlations remain one of major challenges.

With the significant advancements of technologies in identifying genetic predispositions, especially the next-generation sequencing technology (4–9), the number of IRD-related genes and mutations has increased dramatically to date. Thus, it is crucial to integrate the existing data and present an organized comprehensive mutation repository, which could greatly facilitate the genetic counseling, diagnosis and gene therapy. To date, three databases related to retinal diseases, KMeyeDB (10–12), the Mutation Database of Retina International (http://www.retina-internatinal.org/sci-news/mutation.htm) and RetNet (https://sph.uth.edu/retnet/), have been constructed, which contains 121 genes, 176 genes and 202 genes, respectively. However, with the growing number of IRD-related genes and mutations identified recently, the information supplied by these databases is still limited and can hardly meet the demand of researchers and clinicians (4).

In this study, ‘RetinoGenetics’, which contains more genes and mutations, was developed to provide full-scale mutation information and extensive annotations for genetic retinal disorders. Firstly, we retrospectively extracted the basic information (gene symbol, ethnicity, disease, coding sequence change, which known as CDS change, PubMed ID, etc.) for each mutation from the publications. Secondly, informative annotations were performed, which include general gene information, Gene Ontology (GO), KEGG pathways, protein–protein interaction (PPI), mutation exegeses and gene–disease network. As a result, 186 genes, 3143 single nucleotide variations (SNVs), 1127 insertion or deletion (InDels) and 164 different phenotypes were included. In general, the ‘RetinoGenetics’ is constructed to be a well-organized and comprehensive mutation resource for clinicians and researchers on hereditary retinal diseases.

## Methods

### Data collection and content

To obtain a complete list of genes and mutations associated with IRD, we performed comprehensive searches for the IRD-related publications. Initially, we retrospectively queried the PubMed database (http://www.ncbi.nih.gov/pubmed) for genes retrieved in Entrez with terms ‘gene symbol [Title/Abstract] AND mutation [Title/Abstract] OR variant [Title/Abstract] OR variation [Title/Abstract]’. Additionally, other three databases were taken as supplementary sources, which are Leiden Open Variation Database 2 (LOVD2) (13), the Mutation Database of Retina International (http://www.retina-internatinal.org/sci-news/mutation.htm) and Online Mendelian Inheritance in Man (14). In LOVD2, the mutations predicted to be benign or did not segregate with phenotype were excluded.

Consequently, >1000 publications started from 1992 were obtained. After manually screening the abstracts of these articles, we excluded the reviews and those studying other diseases rather than IRD and eventually retained 934 publications that contain IRD-related genes and candidate mutations. All the information, including PubMed ID, ethnicity, disease name, gene symbol and, most importantly, CDS change, was extracted through full-text publication reading and curation and double-checked manually. To obtain correct genomic coordinate for each variant using in-house program, the mutations with only amino acid changes but no accurate CDS changes were excluded. Finally, ‘RetinoGenetics’ database contains 186 genes, 3143 SNVs, 1127 InDels, 164 clinical phenotypes and their detailed information from 934 publications published before 28 February 2013.

### Functional analysis

**Figure 1.**
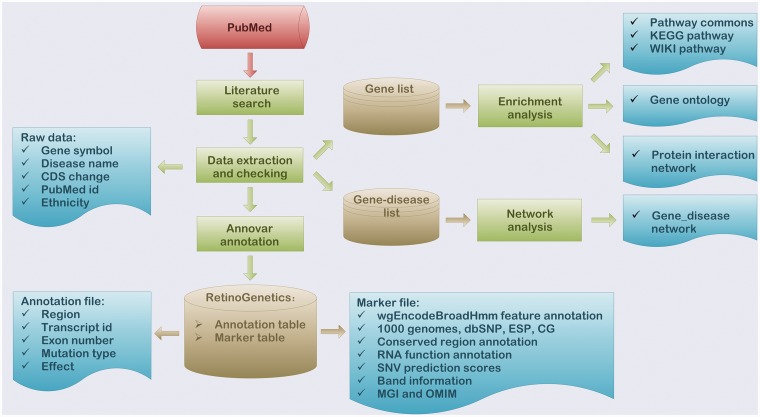
Flowchart of the procedure for ‘RetinoGenetics’. ‘RetinoGenetics’ mainly consists of three parts: (i) data extraction based on publication search, (ii) mutation annotation of all variants and genes using ANNOVAR and (iii) enrichment analysis with all IRD-related genes by WebGestalt and network analysis graphically showing intrinsic relations between genes and phenotypes.

To deeper interpret the function of collected data, a series of functional analyses were performed, which included enrichment analysis, mutation annotation, gene–disease network construction and mutation spectrum (Figure 1).

#### Enrichment analysis

To extensively annotate the IRD-genes, we provided GO annotations, KEGG pathways, WIKI pathways, pathway commons and PPI information and made a rough summary for genes stored in ‘RetinoGenetics’ using WebGestalt (15, 16). Researchers may link to the ‘Gene report’ page to see whether the gene of interest is involved in any pathways, GO terms or PPIs. The gene with the highest number of mutations in ‘RetinoGenetics’ is ‘ABCA4’, with 844 mutations, followed by ‘RPGR’ with 365, ‘USH2A’ with 347 and ‘WFS1’ with 127 mutations. Interestingly, these four genes are commonly involved in three biological processes with the most statistically significant *P*-values of all GO terms according to the GO annotations, namely, sensory perception of light stimulus (*P*-value: 1.4E-142) (17), visual perception (*P*-value: 6.26E-141) (17) and sensory perception (*P*-value: 6.18E-103) (18). In addition, pathways such as rod visual signal transduction (*P*-value: 5.89e-31) and cone visual signal transduction (*P*-value: 2.03E-26) (19) were also statistically significantly enriched. Over and above, ‘H-sapiens_Module_43’, which ranks the first with the *P*-value of 5.88E-17 among all PPIs enriched, was annotated as participating in the biological process of cilium morphogenesis (*P*-value: 7.44E-15) (20, 21). The above analysis is consistent with previous findings that sensory perception of light stimulus, visual perception, sensory perception, visual signal transduction and cilium morphogenesis are related to retinal degeneration.

#### Mutation annotation

To facilitate the users to assess all the information regarding the mutation of interest, detailed annotation of the mutations were performed. Firstly, the coordinates of mutations on reference genes (such as NM_012469, c.2185C>T) were converted to the corresponding coordinates on human reference genome hg19 (UCSC, such as chr20:62658491), whose genomic coordinates were retrieved by UCSC Genome Browser (22). The in-house program that converts coordinates from CDS to genome was written in Perl and was available on the Web site. Secondly, the general annotation of mutations, such as the locations (exonic, intronic, intergenic, region, etc.) and effects on protein coding (synonymous, missense, nonsense, frameshift, etc.), was performed by ANNOVAR (23). Additionally, to provide more detail information about each variation, another 25 databases or data sets were reflinked and annotated, such as dbSNP (24) and 1000 Genomes Project (25).

#### Gene–disease network and mutation spectrum

To facilitate the exploration of the exceptional genetic heterogeneity of hereditary retinal diseases, gene–disease network, drawn by scalable vector graphics (SVG), was completed to graphically display intrinsic relations between IRD and causative genes. A graphical gene–disease network was dynamically generated to provide potential insights and clues for understanding the complex heterogeneity of IRD. Information like number of IRD genes, mutations and common mutations of different disease phenotypes were displayed in the network. Besides, we also used SVG to visualize the mutation distribution in each IRD-gene, with different colors to represent different mutation types or effects, which presented a gene level overview of the summarized mutations. The mutations published more than once in different ethnicities were distinguished by different fonts and colors from those firstly identified.

### Database organization

All the data were stored and managed in a MySQL relational database and were organized in two different tables because of the different output formats of ANNOVAR (23). In addition, GO annotation, KEGG pathways, WIKI pathways, pathway commons and PPIs were stored into separate tables. All data can be freely downloaded from the Web site.

### Web interface and data browse

**Figure 2.**
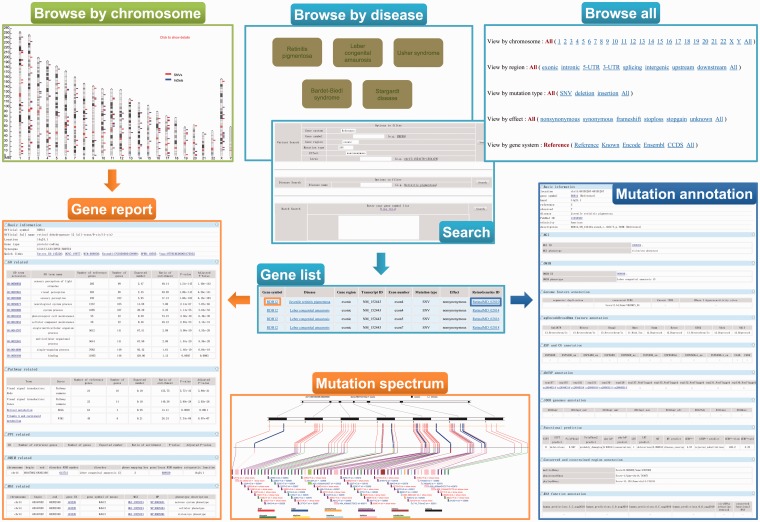
A screenshot of the browse and search tools in ‘RetinoGenetics’. ‘Browse by chromosome’ is used to retrieve all IRD-related genes mapped on chromosomes, and each is linked to ‘Gene report’. ‘Browse by disease’, ‘Browse all’ and ‘Search’ aim to get ‘Gene list’, through which ‘Gene report’ and ‘Mutation annotation’ can be further obtained. ‘Gene report’ lists a detailed summary of the gene including ‘Mutation spectrum’, whereas ‘Mutation annotation’ displays information like location, clinical phenotype, functional prediction and details in existing databases.

A user-friendly Web interface for browsing and searching has been designed and implemented for ‘RetinoGenetics’ by PHP and JavaScript running on an Apache HTTP server. The Web client is implemented independently of the operating system and has been successfully tested with Microsoft Internet Explorer 10, Firefox 2/3, Google Chrome 24.0 and Safari 6.02. To facilitate users to browse the data, three different approaches are provided: (i) browse all, (ii) browse by disease and (iii) browse by chromosome (Figure 2). The ‘browse all’ provides five options (chromosome, gene region, mutation type, effect and gene system) for users to retrieve the information of mutations of interest conveniently. Meanwhile, top seven hereditary retinal diseases with the highest number of related mutations are presented. The genes and mutations related to this disease can be easily retrieved by selecting a listed disease. Additionally, users can browse the entry by chromosome in a graphical way, in which all the variants are mapped on the chromosomes and linked to gene information page.

To ensure a user-friendly Web interface, the final entry does not differ, although users browse in different ways. In each entry, a summary of information about the variant is included, and two hyperlinks are provided to get detailed information about the gene symbol or the variant. To access pathways, GO terms, PPIs, mutation spectrum and gene–disease network specific to the gene of interest, users may link to the gene symbol hyperlink to view details. Function annotations performed by ANNOVAR are displayed in the detail page of RetinoGenetics ID. Gene–disease network provides an easily accessible picture of the IRD-gene networking in a graphical view.

### Data search

‘RetinoGenetics’ provides both key word and sequence search for the data. Firstly, a quick search box can be found on each page for searching by gene symbol or RetinoGenetics ID. Secondly, three search modules (‘Variant Search’, ‘Disease Search’ and ‘Batch Search’) in advanced search page were incorporated to allows users to (i) search by specifying options like gene region, mutation type, effect or locus of mutations, (ii) investigate the mutations of a specified disease, which was not listed in the top seven inherited retinal diseases and (iii) search mutation data of more than one gene, and a gene symbol list will be needed to achieve this search. Finally, a BLAST search against the nucleotide or protein sequences of all genes in ‘RetinoGenetics’ is also available.

## Results and Discussion

In this study, we developed a comprehensive mutation database, ‘RetinoGenetics’, for the genetic information related to IRD, which covers a broad range of data including lists of genes, mutations and diseases, GO terms, pathways and PPIs. ‘RetinoGenetics’ integrated a broad range of data types associated with IRD through in-depth mining of 934 publications and extensive ‘in silico’ functional analyses. It supplies intuitive Web interface to browse or search the data conveniently. With the help of advanced search functions, convenient browsing ways and intuitive graphical displays, the ‘RetinoGenetics’ database could serve as a valuable resource for unveiling the genetic basis of IRD.

Although the development of the next-generation sequencing in recent years has enhanced our knowledge on ophthalmic genetics, there is still about half proportion of IRD patients with unidentified genetic mutations (26–28), which implicates undiscovered disease genes in these patients. The functions of the known IRD genes are mostly involved in specific GO items, such as retinal rod cell development, retinal cone cell development and visual perception. Many of the novel or unrecognized genes actually interact with known genes in IRD (29–32). Taken together, these data suggest that these networked genes potentially interact with key IRD genes and could induce the disease state. In this study, functional analyses were performed including enriched GO terms, pathways and PPIs. If researchers are interested in a specific IRD gene, they may link to the ‘Gene report’ page to see whether the gene is involved in any retinal-related pathways, GO terms or PPIs. These data would be important and useful for revealing novel IRD-related genes. For example, we found that four genes with highest number of mutations in RetinoGenetics are commonly involved in sensory perception of light stimulus, visual perception, sensory perception and visual signal transduction were significantly enriched. These data not only further demonstrated that these four genes play important roles in IRD but also indicated that these GO items might be the key pathways. Therefore, researchers could focus on the other unknown genes, which were involved in the same GO item.

The number of mutations discovered underlying IRD is expected to keep increasing owing to the next-generation sequencing technology. Automatic IRD-related publications mining methods will be used in the updating of data in ‘RetinoGenetics’. Continuous efforts will be made to (i) collect the latest mutation data related to IRD, (ii) perform extended functional analysis based on the updated data sets and (iii) improve the gene–disease network, mutation spectrum and other database functionalities. Additionally, ‘RetinoGenetics’ is planning to support new data to be submitted directly to ‘RetinoGenetics’ to keep it up-to-date and comprehensive. Taken together, we believe that ‘RetinoGenetics’ will be beneficial to our better understanding of the complex heterogeneity of IRD and hope that the continued efforts to improve ‘RetinoGenetics’ will ultimately help the improvement of disease diagnosis and treatment.
