# ‘RetinoGenetics’: a comprehensive mutation database for genes related to inherited retinal degeneration

**DOI:** 10.1093/database/bau071

**Published:** 2014-07-09

**Authors:** Xia Ran, Wei-Jun Cai, Xiu-Feng Huang, Qi Liu, Fan Lu, Jia Qu, Jinyu Wu, Zi-Bing Jin

*Database* 2014 (2014), doi:10.1093/database/bau047, pp. 1–6

The author incorrectly supplied the URL for the Mutation Database of Retina International. The correct link is http://www.retina-international.org/.

The author apologizes for this mistake.

